# Knee and Ankle Arthroplasty in Hemophilia

**DOI:** 10.3390/jcm6110107

**Published:** 2017-11-22

**Authors:** Luigi Piero Solimeno, Gianluigi Pasta

**Affiliations:** Division of Orthopaedic Surgery and Traumatology, Fondazione IRCCS Ca’ Granda, Ospedale Maggiore Policlinico, Via F. Sforza, 28, 20122 Milan, Italy; traumatologia_urgenza@policlinico.mi.it

**Keywords:** hemophilia, knee, ankle, arthroplasty, replacement

## Abstract

Today, major surgical procedures can be safely performed in hemophilic patients with chronic arthropathy, using available factor concentrates. In this setting, total knee replacement is considered the “gold standard”, while the use of total ankle replacement is still debated. Indeed, the unsatisfactory results obtained with the previous available design of implants did not raise enthusiasm as knee or hip replacement. Recently, the introduction of new implant designs and better reported outcomes have renewed the interest in total ankle replacement in people with hemophilia. In this review, the role of replacement surgery in the treatment of chronic hemophilic arthropathy will be described.

## 1. Introduction

“Target joints” are the result of recurrent bleeding into joints causing hypertrophic and hypervascularized synovitis, increasing the joint’s susceptibility to further bleeding [1,2]. Repeated hemarthroses with iron deposit in the synovium promote a cytokine-mediated inflammatory response leading to the progressive destruction of both cartilage and bone [3,4]. Chronic hemophilic arthropathy is the final result of these changes, and it is characterized by pain, stiffness and deformity [5]. Elbows, knees and ankles are the joints most often affected [6]. Nowadays, total knee replacement should be considered the “gold standard” for the patient with chronic hemophilic arthropathy of the knee conversely, the use of ankle replacement with the same indication is still debated. Indeed, the unsatisfactory results obtained with the previous available design of implants did not raise enthusiasm as knee or hip replacement. Recently, the introduction of new implant designs and better reported outcomes have renewed the interest in total ankle replacement in people with hemophilia. In this review, the role of replacement surgery in the treatment of chronic hemophilic arthropathy will be described.

## 2. Knee Arthroplasty

### 2.1. Surgical Indications

The presence of severe disabling pain is the primary indication for knee replacement in a person with hemophilia. Contributing indications are deformity and poor functional range of motion that in severe cases can be considered as the main indications even if severe pain is not present. Restricted range of motion is commonly observed in hemophilia patients, and regaining a functional range of motion after replacement surgery is always a great challenge. There is sometimes a reluctance to perform joint replacement in young patients. However, if a total joint replacement is indicated (e.g., pain, functional impairment, mal-alignment), there is no gain to be made in waiting, and patient age should not be the limiting factor. It is important to explain to patients that although they may not experience pain, surgery may still be indicated due to functional impairment and the need to think of their future mobility and joint function.

### 2.2. Pre-Operative Assessment

A full musculoskeletal assessment is necessary to evaluate a person with hemophilia who is a candidate for replacement surgery. The patient’s gait and posture, the ability to perform the daily life activities have to be observed with associated deformities at the hip, foot and ankle in the affected and the opposite lower limb. Multiple joint involvement is commonly observed and must be taken into consideration in order to have the right surgical planning. Before performing surgical procedures in a patient with hemophilia, a thorough medical evaluation should be performed. Baseline assessment includes factor levels, testing for the presence of inhibitors, liver function studies and viral studies, which should include HIV and hepatitis C.

Pain, restricted range of motion and deformities are the main characteristics of chronic hemophilic arthropathy of the knee succeeding severe functional impairment. Flexion contracture is the most common deformity observed as the result of the flexed position assumed in order to reduce pain during recurrent hemarthroses by the patient with hemophilia. The progression of the disease with severe cartilage and subchondral bone damage can lead the knee to a fixed position of flexion.

External rotation and posterior subluxation of the tibia on the femur with valgus deformity are commonly associated with flexion contracture. Retraction of the soft tissue, severe destruction of articular cartilage and subchondral bone with cysts and erosions and asymmetric bone loss are generally associated with deformity.

The clinical examination of the knee should include the measurement of both passive and active range of motion. In the case of deformity, it is necessary to determine if it is passively correctable or whether it is fixed. The assessment of quadriceps atrophy, the severity of soft tissues contracture and the position and mobility of the patella should be part of clinical evaluation.

In order to correctly assess deformity and associated bone loss, pre-operative radiographic planning is needed. A frequent feature of chronic hemophilic arthropathy is the presence of large subchondral bone cysts or poor bone quality of the femur or tibia, which may need bone graft or prosthetic augmentation. Additional common radiological findings are abnormalities in the position of the patella and of the patellar groove. An anterior-posterior and lateral standing view and a notch and sunrise view, of both knees, are necessary. Moreover, it is preferable to add an antero-posterior view on a long film, which includes the hip to the ankle, and a CT scan to complete the evaluation of bone loss.

It should be clear that pain relief is the main and easiest goal to obtain after the surgical procedure. The achievement of the functional arc of movement will be the hardest goal to obtain for the patient, and the surgeon has to explain it extensively in the pre-operative period [7]. It is mandatory for all the involved actors (i.e., the patient, surgeon and physiotherapists) to understand that the result in term of range of motion improvement is not always possible. Moreover, it is important to explain that desirable post-operative arc of motion means zero degrees extension to the best level of flexion that the patient can reach. Usually, the amount of flexion obtained at surgery and maintained in the post-operative period is related to the status of the extensor mechanism and the level of patient cooperation.

### 2.3. Surgical Technique

Antibiotic prophylaxis starts pre-operatively with the first dose administered thirty minutes prior to surgery. The procedure is routinely performed under tourniquet control prior to the implantation of the components when it will be deflated in order to have a full view of the joint to achieve an accurate hemostasis. Hence, the tourniquet can be reinflated when the components have to be cemented. The use of cement with antibiotic is generally advised.

After the final implantation, the joint is irrigated, and a drain is placed and kept in place for 24–48 h postoperatively. The capsule and the extensor mechanism are closed with non-absorbable sutures with the knee in a flexed position. In order to test the suture and check if the extensor mechanism tracks centrally, a full passive range of motion of the knee is performed. Metal staples are advised to close the skin.

### 2.4. Post-Operative Period

In the first 24–48 h post-operatively, a compressive bandage or a splint could be used to maintain extension of the knee. Subsequently, flexion could start with the supervision of an experienced physiotherapist who is not excessively fearful of causing bleedings and can utilize the appropriate amount of force in passive and assisted active range of motion. Nevertheless, attention should be focused on maintaining full extension considering the tendency of the patient to keep the knee in a flexed position in order to reduce pain. The use of the continuous passive motion (CPM) machine is generally advised, and it is helpful in order to gain flexion, while it is difficult to obtain extension [8]. Initially, all the efforts should be focused on gaining full active knee extension, and the CPM machine should be used intermittently on a daily basis. A prolonged rehabilitation period is usually necessary for the patient to obtain complete recovery, and this includes physical medicine and rehabilitation twice daily and five days a week for the first 3–4 weeks after surgery. A clotting factor level of 30% prior to each session is generally enough to safely perform the rehabilitation program. If the results are encouraging, physical medicine and rehabilitation could be reduced to three days a week and continued for an additional period of six to nine weeks. Unfortunately, despite a post-operative continuous passive motion, rigorous physical medicine, rehabilitation and patient cooperation, a progressive loss of range of motion can occur related to the formation of new fibrous tissue. The patient gradually loses function to end with a very restricted range of motion and in some cases fibrous ankylosis [9].

### 2.5. Results and Complications

Total knee replacement (TKR) is considered the gold standard for the treatment of end stage chronic arthropathy in hemophilic patients [10,11]. Unfortunately, the reported prevalence of infection ranges from 0 to 17%, which is much higher than the prevalence of 1–2% observed after TKRs in the non-hemophilic population, and the rate of prosthetic survival is 90% after five years [12,13,14]. Recently, the mean rate of infection was reported as 6.9, ranging from 1.4 to 11.4% [10]. In order to obtain a decrease of infection rate with similar outcomes to that of total knee arthroplasty in the general population, the maintenance of a high level of clotting factor replacement throughout wound healing has been supported [15]. The effectiveness of the use of antibiotic-loaded cement to prevent the onset of infection after primary total knee replacement in patients with hemophilia is still controversial [16].

Different modalities of treatment are available to treat an infectious complication of a TKR: long-term antibiotics, debridement with retention of the prosthesis, one- or two-stage revision surgery and arthrodesis. One-stage revision should be limited to those patients with a high risk for anesthesiology and when the organism cause of the infection is single and having low or negligible virulence (i.e., methicillin-sensitive coagulase-negative staphylococci). A rate up to 80% of good results has been reported for the revision of total hip replacement secondary to infection [17]. Nevertheless, the two-stage revision technique is the most used, and it has been associated with the most successful results in order to treat infection after replacement surgery [18].

## 3. Ankle Arthroplasty

### 3.1. Surgical Indications

Ankle arthroplasty is indicated in patients with persistent and severe pain limiting daily activities, and restricted range of motion and deformity have to be considered secondly. Active infection, poor skin status (i.e., multiple scars, varicose ulcers), severe bone defect and severe unreconstructed ligament laxity are absolute contraindications.

### 3.2. Pre-Operative Assessment

A detailed history and thorough physical examination (muscle function and strength, range of motion, tendon excursion, standing lower limb alignment, gait) are necessary.

In order to obtain a complete clinical assessment, it is always important to remember that the ankle is constituted by the tibiotalar joint and the subtalar joint and that in patient with hemophilia could be both involved in and related to the clinical symptoms reported by the patient. The limitation of dorsiflexion or a fixed plantar flexion is the most commonly-reported functional sign associated with the presence of anterior osteophytes in the tibiotalar joint and secondary retraction of Achilles tendon. Moreover, the flattening of talar dome and the involvement of subtalar joint lead to a flat foot with a valgus deviation of the hindfoot [19].

Radiologic assessment has to include a static study of both lower limbs under loading, antero-posterior and lateral ankle and foot views and sometimes dynamic X-ray (full extension and flexion). Computed tomography should be indicated in order to assess subchondral cysts and bone loss.

### 3.3. Surgical Technique

Antibiotic prophylaxis is administered at induction of anesthesia before the application of the tourniquet. An anterior longitudinal surgical approach is generally used, and the extensor retinaculum is incised in line with the skin incision as the joint capsule. Anterior osteophyte removal and synovectomy are performed prior to bone cut. After the insertion of trial components, the positioning, the mobility and the stability of the joint are checked by X-ray with an image intensifier. The size and the thickness of the polyethylene bearing are selected by performing successive tests. Additional surgeries as Achilles tendon lengthening or arthrodesis are frequently performed [5]. The wound is sutured, and a drain is left in place 24–48 h after the procedure.

### 3.4. Post-Operative Period

In order to facilitate the wound care and early rehabilitation, a removable boot is used. The rehabilitation program is started a few days after surgery with passive mobilization. Weight-bearing and active motion are allowed 15–30 days after surgery, and rehabilitation should be performed on a daily basis for the first 45–60 days, preferably in a specialist physical medicine and rehabilitation department. Normal physical activity could be re-started after three months, approximately.

### 3.5. Results and Complications

Reported clinical results of ankle arthroplasty in hemophilic patients [20,21,22] are encouraging with relief of pain and improved (although limited) joint mobility in the vast majority of patients. 

In the largest published series [23,24,25], no intraoperative or perioperative complications were observed, and wound healing occurred within two weeks in all patients. However, Strauss et al. reported [25] two cases (2/11) of early periprosthetic infection requiring implant removal, and Asencio et al. [23] reported two cases of revision surgery (2/32) for fixation failure and worsening pain.

Despite the encouraging aforementioned experiences, the use of joint replacement for the treatment of end stage hemophilic ankle arthropathy is still controversial [26,27]. Large prospective studies with longer follow-up results are needed in order to confirm the effectiveness and safety of this surgical technique and to validate indications and timing.

## 4. Conclusions

Although successful reported experiences are increasing worldwide, joint replacement surgery in patients with hemophilia is still a challenge for orthopedic surgeons in comparison with the general population. The presence of different surgical indications, the need for different surgical techniques and dedicated multidisciplinary post-operative care and the higher rate of complication have to be clearly highlighted. Moreover, patients with hemophilia have to be considered active members of the treatment team and should be clearly informed about the risk-benefit ratio of each surgical procedure taking into account functional improvement, quality of life amelioration and risk of complications.

In light of these considerations, in order to achieve good outcomes after replacement surgery in patients with hemophilia, the availability of skilled orthopedic surgeons and physiotherapists is crucial.
